# Draft genome sequence of *Acinetobacter soli* CELK8 isolated from the taro (*Colocasia esculenta*) rhizosphere

**DOI:** 10.1128/mra.00831-26

**Published:** 2026-10-01

**Authors:** Sanjoy Kumar Mukharjee, Md. Faruk Hasan, Biswanath Sikdar

**Affiliations:** 1 Department of Microbiology, University of Rajshahi 118869, Rajshahi, Bangladesh; 2 Department of Microbiology, Noakhali Science and Technology University 378872, Noakhali, Bangladesh; 3 Department of Genetic Engineering and Biotechnology, University of Rajshahi 118869, Rajshahi, Bangladesh; University of Strathclyde, Glasgow, United Kingdom

**Keywords:** whole-genome sequencing, *Acinetobacter soli*, taro, rhizosphere, plant growth promotion, biosynthetic gene clusters

## Abstract

Draft genome sequencing of *Acinetobacter soli* CELK8, isolated from the taro rhizosphere in Bangladesh, revealed a 3,478,463 bp genome assembled into 126 contigs with 43.0% GC content and 126.5× coverage. Genome analysis predicted genes linked to plant growth-promoting (PGP) traits and biosynthetic gene clusters, highlighting its multi-trait PGP potential.

## ANNOUNCEMENT

*Acinetobacter soli*, originally described from soil (1), has been isolated from diverse environments (2–6), but its functions in plant-associated habitats remain poorly understood. Here, we report the draft genome sequence of *A. soli* strain CELK8, isolated from the rhizosphere of taro (*Colocasia esculenta*). This strain was previously characterized during the screening of taro rhizobacterial isolates for plant growth-promoting traits, abiotic stress tolerance, and antifungal activity (7).

Taro rhizosphere soil was collected from a single healthy taro plant grown in Lakshmipur District, Bangladesh (22° 55.03′ N, 90° 52.43′ E). Approximately 10 g of root-adhering soil was collected after uprooting the plant. The soil was suspended in 90 mL sterile distilled water and stirred for 30 min. The supernatant was serially diluted (10⁻¹ to 10⁻⁶) without filtration. Aliquots (0.1 mL) from each dilution were spread directly onto nutrient agar (NA) plates (HiMedia Laboratories, India) and incubated at 28 ± 2°C for 48 h under aerobic conditions. A pure culture of isolate CELK8 was obtained by repeated streaking on NA plates under the same conditions until a morphologically uniform colony was obtained.

For whole-genome sequencing, the pure culture was grown in nutrient broth under similar incubation conditions and submitted to the icddr,b Genome Center, Mohakhali, Dhaka 1212, Bangladesh, where genomic DNA was extracted using the Wizard Genomic DNA Purification Kit (cat. A1620; Promega, Madison, WI, USA). Libraries were prepared with the Illumina DNA Prep Kit (Illumina, San Diego, CA, USA), and paired-end (2 × 150 bp) sequencing was performed on the Illumina NextSeq 2000 platform, generating 859,245 raw read pairs. Sequence reads were quality assessed using FastQC v0.12.1 (8) and trimmed using FastP v1.0.1 (9). *De novo* genome assembly was performed with SPAdes v4.2.0 (10), generating an initial assembly of 146 contigs. Contigs shorter than 200 bp were removed before downstream analyses. Assembly quality was evaluated using QUAST v5.3.0 (11). Taxonomic assignment was confirmed by average nucleotide identity (ANI) using JSpeciesWS v5.0.3 (12) and digital DNA-DNA hybridization (dDDH) using Type (Strain) Genome Server v415 (13). Genome annotation was carried out using the NCBI Prokaryotic Genome Annotation Pipeline (PGAP) v6.10 (14) and the Rapid Annotations using Subsystems Technology (RAST) server with the RASTtk pipeline (15). Secondary metabolite biosynthetic gene clusters (BGCs) were predicted using antiSMASH v8.0.4 (16).

The final draft genome assembly comprised 126 contigs (>200 bp), with a total length of 3,478,463 bp, an N50 of 288,588 bp, and a GC content of 43.0%. PGAP annotation predicted 3,205 protein-coding sequences (CDSs), 62 tRNA genes, 8 rRNA genes, and 1 CRISPR array. The ANI of 98.5% against *A. soli* M3-1-68 (CP073241.1) and a dDDH value of 88.5% confirmed its identity as *A. soli*. RAST assigned 302 subsystems, predominantly amino acids and derivatives (266 CDSs), protein metabolism (203 CDSs), and carbohydrates (189 CDSs). AntiSMASH predicted eight BGCs for secondary metabolite production, showing similarity to known clusters responsible for bacillomycin D, aryl polyene from *Vibrio fischeri*, fimsbactin A, and berninamycin-related compounds.

## Data Availability

This Whole Genome Shotgun project has been deposited in DDBJ/ENA/GenBank under the accession JBTFFS000000000. The BioSample and BioProject accession numbers associated with this study are SAMN54323834 and PRJNA1344764, respectively. The raw sequencing data have been deposited in the Sequence Read Archive under accession number SRR36609844. The complete RAST annotation output and antiSMASH v8.0.4 prediction results are available through Zenodo (DOI: https://doi.org/10.5281/zenodo.22308944).
